# Quantification of the oxygen uptake rate in a dissolved oxygen controlled oscillating jet‐driven microbioreactor

**DOI:** 10.1002/jctb.4833

**Published:** 2016-01-12

**Authors:** Timothy V Kirk, Marco PC Marques, Anand N Pallipurath Radhakrishnan, Nicolas Szita

**Affiliations:** ^1^ Department of Biochemical EngineeringUniversity College LondonBernard Katz Building, Gordon StreetLondon WC1H 0AHUK

**Keywords:** microbioreactor, Saccharomyces cerevisiae, oxygen uptake rate, oxygen control, mixing, microfluidics

## Abstract

**BACKGROUND:**

Microbioreactors have emerged as a new tool for early bioprocess development. The technology has advanced rapidly in the last decade and obtaining real‐time quantitative data of process variables is nowadays state of the art. In addition, control over process variables has also been achieved. The aim of this study was to build a microbioreactor capable of controlling dissolved oxygen (DO) concentrations and to determine oxygen uptake rate in real time.

**RESULTS:**

An oscillating jet driven, membrane‐aerated microbioreactor was developed without comprising any moving parts. Mixing times of ∼7 s, and k_L_a values of ∼170 h^−1^ were achieved. DO control was achieved by varying the duty cycle of a solenoid microvalve, which changed the gas mixture in the reactor incubator chamber. The microbioreactor supported Saccharomyces cerevisiae growth over 30 h and cell densities of 6.7 g_dcw_ L^−1^. Oxygen uptake rates of ∼34 mmol L^−1^ h^−1^ were achieved.

**CONCLUSION:**

The results highlight the potential of DO‐controlled microbioreactors to obtain real‐time information on oxygen uptake rate, and by extension on cellular metabolism for a variety of cell types over a broad range of processing conditions. © 2015 The Authors. Journal of Chemical Technology & Biotechnology published by John Wiley & Sons Ltd on behalf of Society of Chemical Industry.

## INTRODUCTION

The oxygen uptake rate (OUR) and the specific oxygen uptake rate (sOUR) are frequently employed to characterize fermentation productivity, and to infer the physiological state of cells.^1^, ^2^ Determining the OUR requires precise control over the dissolved oxygen concentration in the bioreactor. This in turn requires that the bioreactor can transfer more oxygen to the cells than is consumed by the cells. Aeration and mixing are therefore of paramount importance to ensure effective gas–liquid mass transfer and homogeneous growth conditions.

In traditional stirred tank reactors (STR), oxygen transfer is typically increased by increasing the airflow rate, the oxygen partial pressure, the angular velocity of the stirrer, or a by combination of these parameters. A number of basic and advanced control strategies have been developed in order to address, for example, variations of the conditions during cultivation, such as dynamically changing media compositions, sensitivity of the cells to high shear stress (as a result of increased agitation), and inherent oxygen probe dynamics.^3^

In shaken systems, despite the significant advances in the monitoring of process variables, process control remains a limitation,^4^ making accurate determination of OUR in such devices challenging. In microtiter plates, this has been circumvented by applying feeding strategies to control pH and provide fed‐batch capabilities.^5^, ^6^ Recently, several automated miniaturized systems based on plate formats have been commercialized, which have the ability to monitor and control in individual wells the pH, DO and temperature (e.g. Pall's Micro‐24 MicroReactor System^7^).

Microbioreactors (µBRs) (or microfluidic bioreactors with sub‐milliliter operating volumes^2^) have emerged as a new tool for early bioprocess development. Interest in them has been driven by their potential to significantly reduce cost and labor *via* automated and parallelized approaches and reduced use of resources.^4^, ^8^, ^9^ µBRs have achieved relevant biomass densities as well as oxygen mass transfer coefficients comparable with bench scale reactors.^1^, ^2^ Monitoring dissolved oxygen concentrations in µBRs in real time using optical sensors is nowadays state of the art.^3^, ^9^, ^10^ Control over oxygen can be achieved by taking advantage of the high diffusivity and solubility of oxygen in poly(dimethylsiloxane) (PDMS).^4^, ^11^ In their pioneering work, Lee *et al.* 12, 13 were able to control oxygen concentrations by pressurizing a PDMS membrane with air (or any other gas mixture). Demonstrating that OUR can be measured in real time will therefore further consolidate the applicability of microbioreactors, and will potentially strengthen the link to conventional stirred tank reactors for scale‐up studies.

The aim of this work was to show that OUR can be determined in real time in an actively mixed µBR with a working volume below 100 µL. *Saccharomyces cerevisiae* was selected as a model system owing to its widespread use in industry. Continuous cultivation of *S*. *cerevisiae* in a µBR with monitoring of the dissolved oxygen was previously reported.^14^ Very recently, monitoring of OUR was demonstrated in a µBR configured as a vertical microbubble column.^15^ Oxygen was measured by inserting a needle‐based optical microsensor into the reactor chamber. In this contribution, we demonstrate the growth of this yeast with real‐time monitoring of OUR in an actively mixed, planar µBR using non‐invasive sensor probes. To achieve this and to facilitate integration of optics and microfluidics, a new mixing approach was developed which uses an eccentrically oscillating jet drive, i.e. does not rely on mechanical moving parts.

## MATERIAL AND METHODS

### Fabrication of microbioreactor

All components of the microbioreactor (µBR) were designed using Solidworks® (Solidworks, Dassault Systems, France). Two rigid 5 mm thick polycarbonate plates (PC, RS Components Ltd, Corby, UK) formed a compressive seal over the µBR body which contained the fluidic channels and the culture chamber, against a microscope slide. The reactor and the sensor recess layer were fabricated from poly(dimethylsiloxane) (PDMS, Sylgard 184, Dow Corning, Belgium); the interconnect layer (to connect with tubing) was fabricated from Silastic T‐4 rubber (Dow Corning, Belgium) while the clamping system was fabricated from PC.

The PC was machined with a micromilling machine (Folken IND, USA), using a 500 µm square end mill with a spindle speed of 12 000 rpm. The PDMS and Silastic T‐4 rubber were cast in micromilled molds, made out of aluminum or poly(methyl methacrylate) (PMMA, Plexiglas® XT Clear, Nordisk Plast A/S, Denmark), respectively. The PDMS aeration membrane was spin‐coated (P6708D, Specialty Coating Systems, Indianapolis, USA) onto a silanized 4″ silicon wafer (Prolog Semicor Ltd, Kiev, Ukraine) and cured at 70°C for 1 h. The PDMS layers were bonded by activation in a plasma cleaner (PDC‐002, Harrick Plasma, USA). The DO sensor spots and Upchurch® connectors were secured in place with a silicone adhesive (692‐542, RS Components Ltd., Corby, UK). Standard connection fittings (P‐221, Upchurch Scientific, USA) were used to attach polytetrafluoroethylene tubing (PTFE, ID 0.75 mm, VWR International Ltd, UK). Membrane thickness and surface roughness were assessed with a Dektak 8P stylus profiler (4 mg contact force, 111 nm resolution setting, Veeco Instruments Inc., USA).

### Monitoring

#### Temperature

Temperature was controlled with a bespoke incubator chamber, heated by a recirculating water bath (Grant Instruments Ltd, Cambridge, UK). A constant current circuit^16^ measured the resistance of NTC thermistors (Betatherm BT310‐03M01 10kΩ, Shrewsbury, USA). This circuit was connected to a data acquisition card (DAQ, PCI‐6221, National Instruments Corporation Ltd, UK). Sampling was performed at 10 Hz. The Steinhart–Hart equation coefficients provided by the supplier were used to calibrate the resistance–temperature relationship for the thermistor.

#### Dissolved oxygen (DO) and optical density (OD)

**Figure 1:**
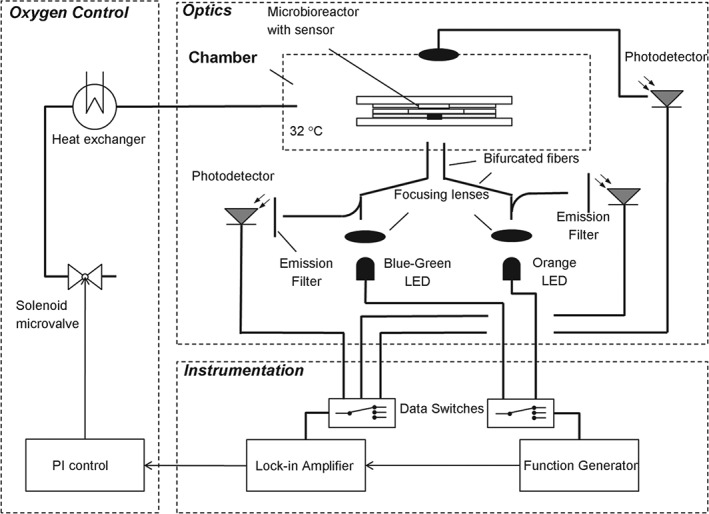
Schematic representation of the experimental setup. The microbioreactor (µBR) is kept at 30°C inside a bespoke incubator chamber. Two optical fibers carry light of different wavelengths to the bottom of the µBR for the measurement of optical density and dissolved oxygen. Photodetectors collect the light that is transmitted or emitted. The voltage signal from the photodetectors is sent to a lock‐in amplifier where it is compared with the signal from the function generator. Dissolved oxygen is controlled by a solenoid microvalve operated as a pulse width modulation (PWM) device to vary the gas mixture in the incubator chamber.

Dissolved oxygen (DO) and optical density (OD) were monitored as presented previously.^17^ Briefly, PSt3 sensor spots (PreSens, Precision Sensing GmbH, Germany) were used to monitor DO. The spots were excited using sinusoidal modulated blue‐green LED (NPE590S, 505 nm, Nichia, Japan). An excitation band‐pass filter (500AF25, Omega Optical Inc., USA) and emission long pass filter (OG590, Schott AG, Germany) were used to separate the excitation and emission signals, respectively, and to minimize cross‐excitation. Data switches (7204‐5, Electro Standards Laboratories, USA) multiplexed the output signal and the input signal of the function generator (FG100, Digimess Instruments Ltd, UK) and the lock‐in amplifier (SR830 DSP, Stanford Research Systems Inc., USA). For OD, an orange LED (LV600‐06V, 600 nm, Epitex, Japan) was employed and a 2 mm diameter cylindrical post was milled in the center of the grid structure at the top of the reactor as a waveguide. All instruments were PC controlled under a LabVIEW routine (National Instruments, USA), which allowed the automated on‐line measurement of both OD and DO (Fig. 1).

#### Calibration of dissolved oxygen sensor and optical density

DO sensor calibration was performed using different concentrations of oxygen in the incubator chamber. Flow meters (variable area rotameter, 03216‐32, Cole‐Parmer, USA) were used to supply air and nitrogen. Mean phase shift values obtained for each oxygen concentration were fitted using a cubic polynomial relation. Calibrations were performed at room temperature and at 30°C. Calibration at 30°C was achieved by passing the gas mixture through a copper tubing heat exchanger connected to the incubator chamber.

OD was calibrated with mono‐disperse polystyrene (PS) microbeads with 5 and 15 µm diameter (Sigma Aldrich, UK), using, diluted solutions of 5 µm PS beads, ranging from 1.4 × 10^6^ to 1.5 × 10^9^ particles mL^−1^, and of 15 µm PS beads, ranging from 5.5 × 10^4^ to 5.6 × 10^7^ particles mL^−1^, respectively. The different bead concentrations were correlated with several dilutions of S. cerevisiae suspensions spectrophotometrically (Ultrospec 3000, GE Healthcare Life Sciences, UK).

### Control algorithms

#### Dissolved oxygen control

DO control was achieved by varying the duty cycle (DC) of a solenoid microvalve (LHDA1211111H, Lee Co., USA) operated as a pulse width modulation (PWM) device at a frequency of 60 Hz. The controller was set up as an anti‐windup PI with the following parameters: P 0.06, I 0.012, 10 Hz DO sampling rate, 60 Hz PMW frequency, 10 Hz output frequency, 0.1% minimum DC and 35% maximum DC. The control algorithm was implemented in LabVIEW (National Instruments, USA)

Filtered air was supplied to the valve's ‘normally closed’ (NC) port, and nitrogen to the ‘normally open’ (NO) port. Main gas supplies were connected via standard microfluidics connectors. The gas mixture was supplied to the incubator chamber at a constant pressure of 1 bar. A schematic of the control system is given in Fig. 1 and further details are given in supporting information (Supporting Information 1).

### Saccharomyces cerevisiae batch culture

Saccharomyces cerevisiae (YSC2, Sigma‐Aldrich, Dorset, UK) inoculum was prepared from mid‐exponential YPD10 medium shake flask cultures. The YPD10 medium consisted of 10 g L ^−1^ yeast extract (Oxoid Ltd, UK), 20 g L^−1^ Bacto™ Peptone (BD Biosciences, UK), 10 g L^−1^ D‐glucose (Fisher Scientific, UK) and 50 mg L^−1^ ampicillin (Sigma‐Aldrich, UK). Growth was performed in 500 mL baffled shaken flasks, with a headspace of 80%, with 50 mm shaking diameter, at a shaking frequency of 200 rpm and 30°C.

The µBR was sterilized by priming with 70% (v/v) ethanol for 30 min (Supporting Information 2), followed by three cycles of flushing/priming with autoclaved deionized (DI) water at intervals of 20 min each. The µBR was filled with YPD10 medium and inoculated with S. cerevisiae. The inoculum syringe was then exchanged with a syringe system containing DI water. Mixing was achieved by withdrawing and infusing a nominal volume of 150 µL with a syringe drive pump (World Precision Instruments, Inc., USA). The pump was operated at a flow rate of 3 mL min^−1^ during withdrawal and 5 mLmin^−1^ during infusion, with a full cycle taking approximately 4 s. End‐point OD values were measured to determine average cell concentration in the µBR. Due to the small volumes involved, the dry weight was determined via the correlation proposed by Oh et al. 18

### Oxygen mass transfer coefficients and oxygen uptake rate

Oxygen volumetric mass transfer coefficient (k_L_a) for the µBR was determined using the dynamic gassing‐out method. Gassing out of oxygen was accomplished by performing concentration step changes in the gas mixture in the incubator chamber (from air to nitrogen). k_L_a was determined from the sensor DO time profile using the following equation:

(1) $$C_{s}\left(t\right)=C^{*}\left[1-\frac{\tau_{m}e^{-t/\tau_{m}}-\tau_{s}e^{-t/\tau_{s}}}{\tau_{m}-\tau_{s}}\right]$$

where C_s_ is the dissolved oxygen concentration over time, C^*^ is the oxygen concentration in equilibrium, t_m_ is the mass transfer time constant, which is equal to 1/k_L_a, and t_s_ is the time constant of the sensor. The oxygen uptake rate (OUR) was determined by the stationary liquid phase balance method. Since the DO is controlled via a PWM gas mixture, the oxygen concentration in equilibrium, C*, was given by the PWM DC, according to:

(2) $$\text{OUR}=k_{L}a\left(DC.C^{*}-C_{O2}\right)$$

Theoretical considerations for oxygen transfer characteristics in microbioreactors have been described in detail in the supplementary information of Kirk and Szita^2^ and further details are given in the supporting information (Supporting Information 1).

### Characterization of mixing

Mixing was characterized through the analysis of a sequence of images of a colored dye mixing with water. This was performed by injecting a solution of 5 g L^−1^ methylene blue (Sigma‐Aldrich, UK) into the sampling port of the µBR. The PDMS aeration membrane was substituted by a glass slide for this characterization. A 25 G syringe needle was inserted into the sampling port. This allowed fluid movement back and forth through the chamber, simulating the effect of the moving membrane. The infuse/withdraw pumping was operated as described in the section Saccharomyces cerevisiae batch culture. Images were captured with a stereomicroscope (Leica Microsystems (UK) Ltd, UK) equipped with a PAL SD video capture device.

Captured images were analyzed using a script written in Python.^19^, ^20^, ^21^, ^22^ The files were scanned and stored in an array, and passed sequentially to a function for image processing. In this function, Python's Imaging Library was used to open the images and the pixel data were copied onto arrays (img_array). Three different arrays were created, one for each color channel in the RGB format: red, green and blue, respectively. A circular array was used to mask the data of the pixels outside the µBR chamber. The center co‐ordinates and diameter of the chamber were obtained through manual measurements using ImageJ.^23^ These values were used to create a mask, which was overlaid on the img_array in order to extract pixel data solely of the µBR chamber area. As a result, the average and the standard deviation of the pixel values were calculated for the masked µBR chamber.

The circular img_array was split across the center of the µBR chamber creating two equal halves. The pixel average and standard deviation of both the halves were calculated, and this routine was repeated three times for all color channels. The mean and standard deviation values of each color channel of all images were stored in another array and later saved as a comma separated value (CSV) file.

Standard deviation of the pixels represents the variation of pixel values within one of the halves of the µBR chamber. Mixing time was, therefore, considered as the point in time (or image) where the standard deviation was within 5%.

## RESULTS AND DISCUSSION

### Microbioreactor design

**Figure 2:**
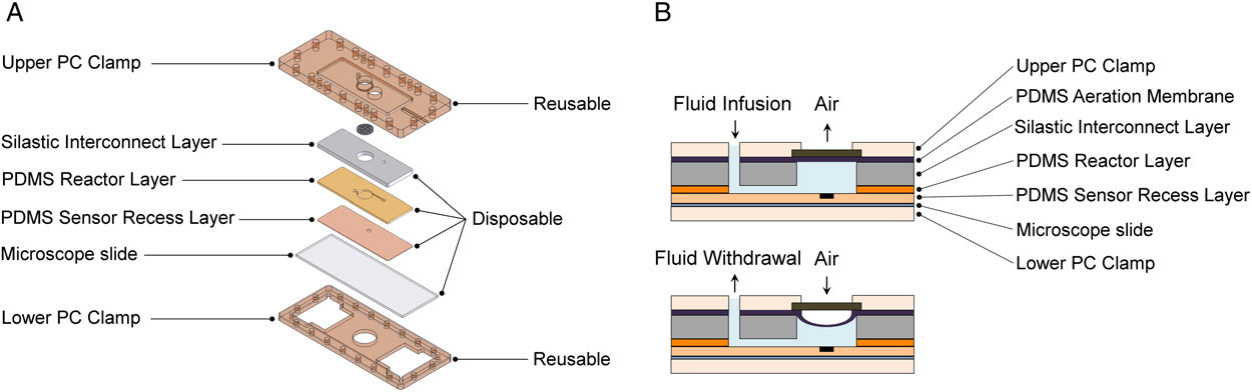
(A) Exploded view of the microbioreactor (µBR) with the three disposable layers, which comprise the reactor structure, and the reusable clamp system. On top of the reactor chamber lays a grid that limits the deformation of the aeration membrane. The disposable layers were made out of poly(dimethylsiloxane) (PDMS) and Silastic T‐4 and the reusable parts were made out of polycarbonate (PC). (B) Schematic representation of the fluid flow induced deformation of the PDMS aeration membrane.

The microbioreactor (µBR) was comprised of a disposable polymeric fluidic chip and a reusable clamp system (Fig. 2). This allowed rapid prototyping and, in addition, reduced sterilization and cleaning loads. There are no moving parts, such as stirrer bars. This also facilitates assembly, as no parts need to be inserted before the reactor chamber is sealed. The polymeric fluidic layer was made out of poly(dimethylsiloxane) (PDMS) and contained a cylindrical chamber (8 mm in diameter, 1 mm deep) with a working volume of 50 µL. The inlet channel (760 µm wide, 500 µm deep) was parallel to the long edge of the chip, connecting with the chamber 3 mm off center, and its floor was flush with the floor of the chamber. The reactor also included a sampling port, positioned on the opposite side of the inlet channel.

Mixing was achieved with an eccentrically oscillating jet, promoted by a single syringe, which infused and withdrew the culture medium to and from the chamber, respectively. For aeration, the chamber was sealed with a 100 µm thick PDMS membrane at the top. The membrane provided also an auxiliary to the mixing itself due to its deformation in the vertical plane, which was caused by the dynamic volume change of the reactor chamber (with each infusion/withdrawal cycle of the syringe drive). DI water was used as the system liquid, i.e. the liquid that transmitted the displacement of the plunger of the syringe to the culture medium in the chamber, enabling in this way the compensation of evaporation.

The disposable PDMS reactor layers were clamped between two layers of PC, which anchored the fluid connectors as well as a grid on top of the reactor chamber. This grid restrained the upward deformation of the aeration membrane. The deformation of this PDMS membrane was controlled by the fluid flow from the infusion‐withdrawal pumping scheme (Fig. 2(B)). This dynamic deformation contributes to the mixing efficiency and promotes medium aeration by increasing the oxygen transfer rate (OTR).

### Characterization of mixing

**Figure 3:**
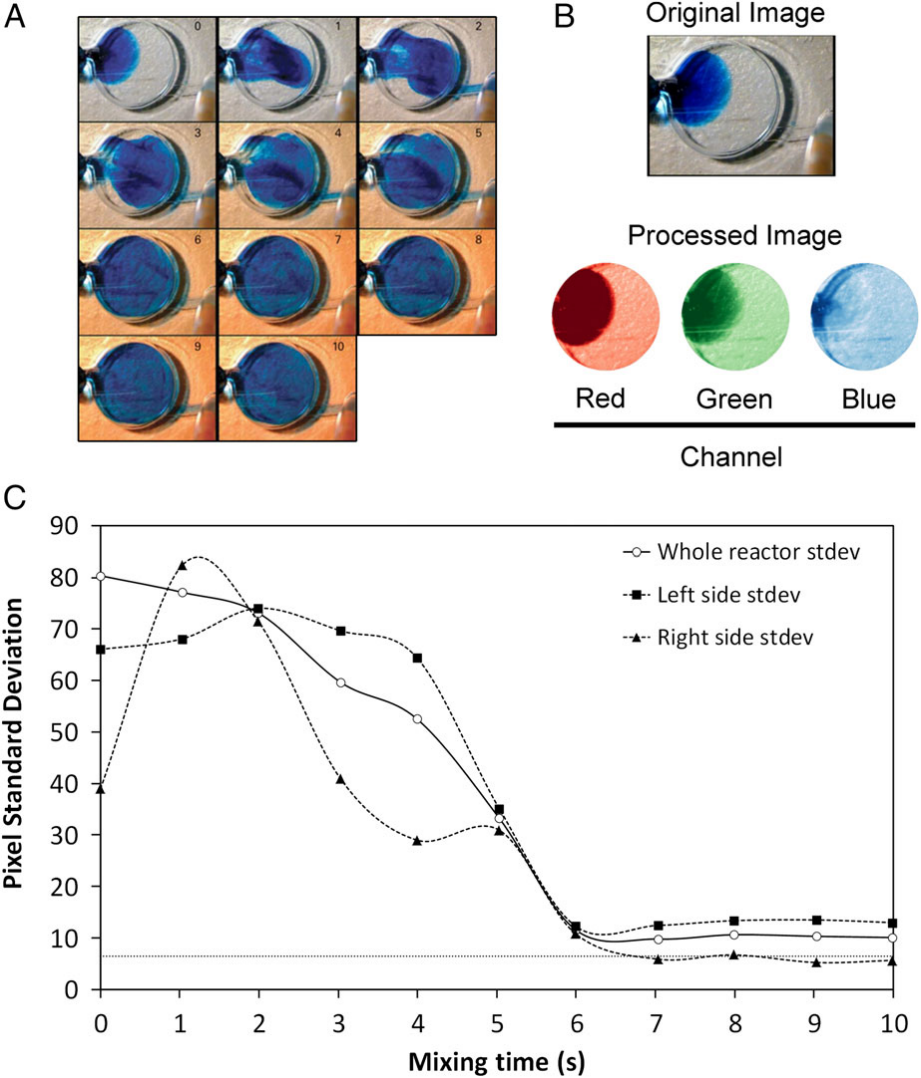
(A) Mixing of 5 µL of a water blue dye solution injected through the sampling port of the microbioreactor (µBR). The infusion rate was set at 3 mL min^−1^ while the withdrawal rate was 5 mL min^−1^ (cycle time of approx. 4 s per cycle). Numbers in the top right corner of frames indicate the elapsed time, in seconds, from the start of the pumping cycle. (B) The original image at t = 0 s, and the masked µBR chamber area for each color channel. Red channel was chosen to compute the mixing time as the blue dye completely transmits blue light. The image pixels of the area outside the µBR chamber were masked using a computational script. (C) Time profile of the variation of pixel standard deviation (stdev). Full squares – left side of the reactor; full triangles – right side of the reactor; open circle – whole µBR chamber. Image insets are the original images for the corresponding data point. Standard deviation is within 5% (dotted line) at 7 s.

In a recycle flow mixing µBR, Li *et al.* 24 obtained a mixing time of approximately 800 s for a flow rate of 20 µL min^−1^. The reactor design was later improved^25^ and a mixing time of 45 s was achieved with a flow rate of 1 mL min^−1^. In our setup, an oscillating flow of infusion rate of 5 mL min^−1^ and withdraw rate of 3 mL min^−1^ (cycle time of approximately 4 s) was used. The intensity of the red color from the water‐soluble blue dye was monitored to characterize mixing. To quantify mixing time, a computational image analysis script, which measured variation in the standard deviation of the pixel intensity over time, was applied. The red channel was chosen from the RGB images as it had the highest absorbance of the three channels for the blue dye (Fig. 3(B)).

The variation in the standard deviation of pixel intensity in the red color channel is shown in Fig. 3(C). A mixing time of 7 s was obtained by calculating the point where the standard deviation is within 5% for the right half of the µBR (as it is on this side that the maximum pixel variation occurred, due to the inlet where the dye was introduced being the farthest away from this area; see the corresponding Materials and methods section).

The results also showed that during the flow oscillation, the dye solution was distributed homogeneously to approximately 90% of the µBR after the first cycle (Fig. 3(A)). This is also evidenced by the standard deviation of the right half of the µBR (Fig. 3(C)), which sharply increases upon infusion of the dye (*t* = 0 to 1 s) and appears to have a stable shoulder region between 4 and 5 s demonstrating the completion of one full cycle. A mixing time of 7 s corresponds to 1.75 cycle times.

### Dissolved oxygen monitoring and control

#### Oxygen mass transfer coefficients

The *k_L_a* was determined by the gassing‐out method, performing step changes in the gas concentration of chamber headspace. DO sensor spot response‐time was taken into account by using a compensation equation and high sampling frequencies (10 Hz). For a membrane thickness of 100 µm, a typical response time would be 4.88 s ± 0.13 s. For the flow rates used in this study, the µBR achieved *k_L_a* values up to 174.0 h^−1^ ± 24.4 h^−1^ (data not shown). This is a clear improvement over diffusion based mixing strategies frequently encountered in µBRs where values in the range of 70 h^−1^ are obtained.^2^, ^17^ The *k_L_a* obtained is in the range of previously published microbioreactors and of traditional bench scale stirred tank reactors.^2^

#### Oxygen control

The dissolved oxygen control system was developed by implementing a proportional‐integral (PI) scheme to control the duty cycle of a solenoid microvalve, and hence the gas mixture output. This scheme was chosen since it was simpler to implement than a PID control and only the gas mixture had to be controlled by a pulse width modulation (PWM) solenoid microvalve.

In their pioneering work Lee *et al.* 12 developed a multiplex system which was composed of eight 100 µL peristaltic mixed reactor chambers bonded to an aeration membrane made from PDMS. Above this membrane, a series of channels were pressurized with either air or another gas mixture. Varying the duty cycle of a solenoid valve set by a PI control scheme allowed significant oxygen control. In a follow‐up study, using a single chemostat/turbidostat microfluidic bioreactor, Lee *et al.* 13 varied the oxygen concentration in the peristaltic mixer actuating gas. A PI control scheme set the duty cycle of the solenoid valve. This improved setup allowed control of oxygen over a three‐week period of continuous fermentation of *Escherichia coli*.

In our work, by controlling the gas mixture in the incubator chamber wherein the µBR is housed, a simpler and more robust control setup can be implemented, while allowing for a simpler reactor design. The control was based on the assumption that the oxygen concentration in the gas phase is in equilibrium with the liquid oxygen concentration at the membrane–liquid interface. This allows the PI algorithm to vary the concentration of oxygen in the incubator chamber until the chosen oxygen concentration set point has been reached or a sustained oscillation occurs. This was achieved by varying the duty cycle of the solenoid valve, in accordance with the difference between the set point and the equilibrium oxygen concentration. The DO concentration in the gas feed was limited to a minimum of 40% of air saturation, which is a reasonable setting in order to avoid sub‐optimal oxygen transfer conditions that would be unsuitable for later scale‐up studies.^26^, ^27^

The control system was characterized prior to the fermentation runs by determining deviations to different imposed set points and by assessing the linearity of the solenoid microvalve. Linearity was observed between the duty cycle and up to the maximum working oxygen concentration, 40% air saturation (Supporting Information 3).

The linearity observed is essential for process control as well as to determine the oxygen concentration in the incubator chamber and ultimately OUR. Improved control performance could probably be achieved if the full range of duty cycles was used. Nonetheless, different gas mixtures (e.g. with higher oxygen concentrations) would be necessary, concomitantly increasing the associated running costs.

#### Temperature control

A variety of methods were trialed for controlling the temperature of the µBR but the best results were obtained with a custom‐built, jacketed aluminum chamber. In addition, the chamber comprised ports that were able to pre‐heat the gas mixture reducing temperature gradients across the µBR.

**Figure 4:**
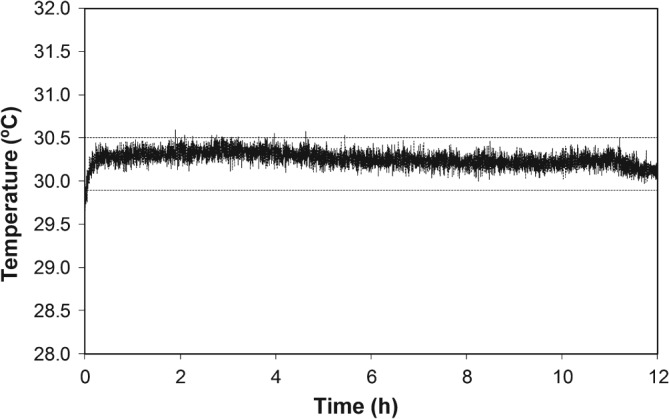
Temperature time‐profile measured at the polydimethylsiloxane (PDMS) reactor layer of the microbioreactor (µBR) during an overnight S. cerevisiae fermentation. The mean temperature after the initial warm‐up period was 30.25°C ± 0.09°C. The dashed lines correspond to a deviation of 1% from the set point of 30.2°C.

Measurements with an NTC thermistor showed a 0.4°C difference across the µBR packaging system when a set point of 30.2°C was chosen. This difference guaranteed a temperature of 30°C inside the µBR chamber (data not shown). Under operational conditions, the incubator maintained an average temperature of 30.25°C for 12 h, measured at the µBR PDMS device base (Fig. 4).

#### Optical density

**Figure 5:**
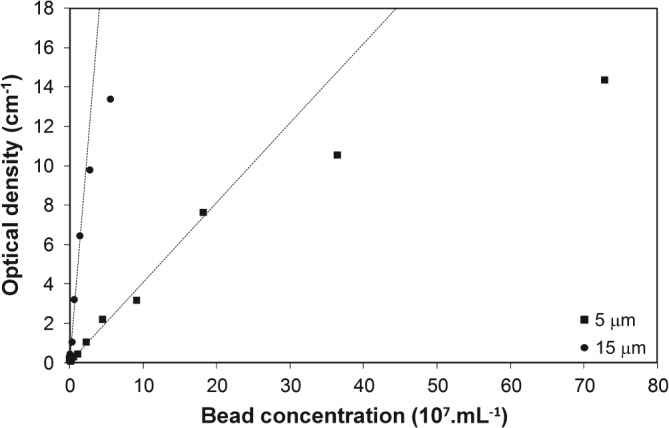
Optical density of different concentrations of 5 and 15 µm beads measured through the 1 mm long path length of the reactor chamber. Dashed lines correspond to the linearity of the optical density measurements.

Calibration was performed using two different diameters of polystyrene (PS) microbeads, 5 and 15 µm, respectively. These diameters were chosen in order to allow a broader range of cell types to be used in the future within the current µBR configuration. Results showed that with 5 µm and 15 µm particles a linear range of up to an OD of ∼ 8 cm^−1^ and ∼ 10 cm^−1^, respectively, was achieved (Fig. 5).

Additional calibrations were performed to assess the influence of the aeration membrane displacement on the OD measurements. For this, different S. cerevisiae suspensions were used and measurements were performed at different locations within the µBR chamber (Table 1).

**Table 1:** Optical density measured in the microbioreactor at different locations with and without the pumping strategy for mixing

Measurement location		OD (cm^−1^)
Without pumping	Centre	3.95
	Perimeter	4.48
With pumping	Centre	4.32
	Perimeter	4.26

### Fermentations with control of dissolved oxygen (DO) and determination of the oxygen uptake rates (OURs)

The feasibility to determine in real time the OUR was evaluated during several DO controlled fermentations of S. cerevisiae. The concentration of glucose used in the runs was set at 10 g L^−1^. This is a comparatively low concentration of glucose. However, the low concentration was chosen to extend the time of fermentation. Ethanol is produced during the fermentation, which is in turn metabolized oxidatively. Therefore, the effect of DO control will be easier to observe than with uncontrolled fermentations.^28^ In addition, as the rates of both ethanol production and consumption are oxygen dependent, increased oxygen availability should result in lower endpoint ethanol concentrations.

**Figure 6:**
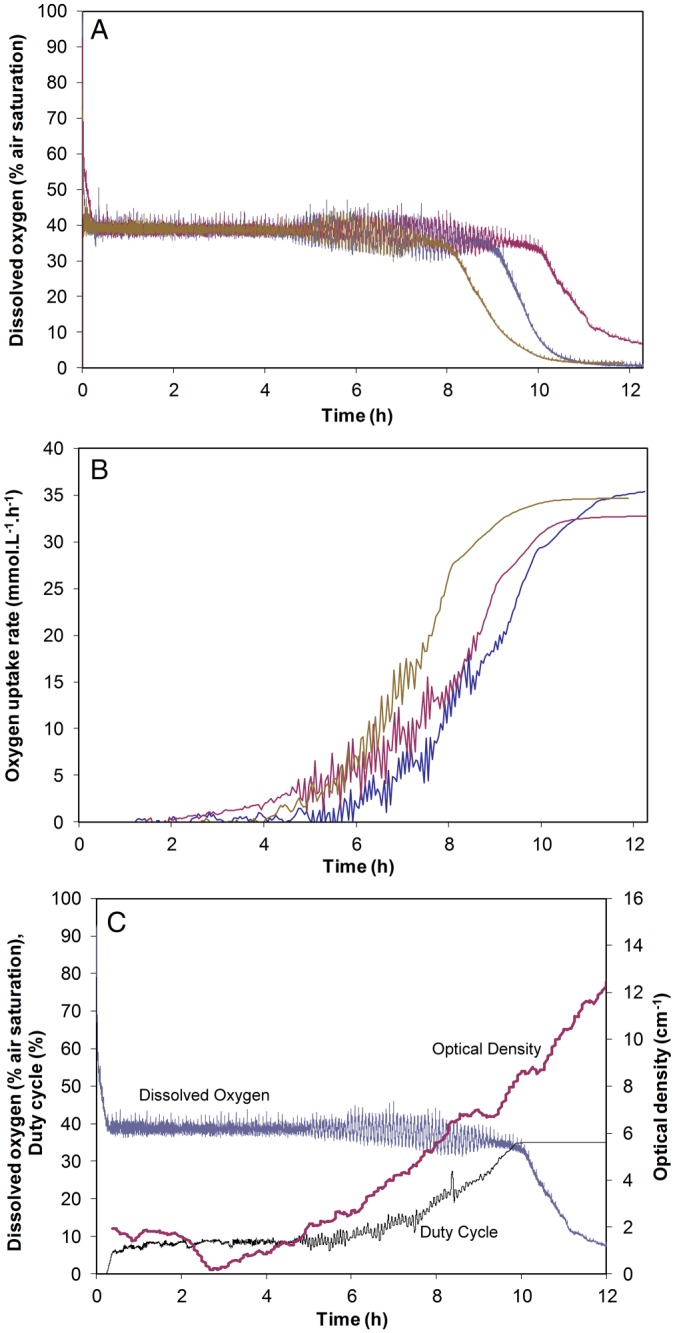
(A) Dissolved oxygen time‐profiles for three independent oxygen controlled Saccharomyces cerevisiae fermentations in YPD media. The range from the mean control period was approx. 1 h. Measurements were performed in independent runs, only changing the disposable layers of the microbioreactor in between runs. (B) Oxygen uptake rate (OUR) for three independent oxygen controlled S. cerevisiae fermentations in YPD media. The OUR was plotted by calculating the mean values of DO and duty cycles (DC) for every 100 samples. The plotted data show the moving averages with a period of 4 data points. Measurements were performed in independent runs changing the disposable layers of the microbioreactor between runs. (C) Dissolved oxygen controlled S. cerevisiae fermentation in YPD media. The oxygen concentration was controlled over a period of 10 h. The end optical density was 9 cm^−1^ which corresponds to an implied k_L_a of 91 h^−1^. The OD curve shows the moving averages with a period of 10 data points.

The implemented control system is robust and repeatable (Fig. 6(A)). Control periods of 8, 9 and 10 h are in the same range (Fig. 6(B)). Comparing the DO control performance of the µBR with other systems is difficult since in the literature, to the best of the authors' knowledge, there are no examples where this type of DO control is used for S. cerevisiae fermentations. Lee et al., 12 for example, ran 8 h fermentations with E. coli reaching end‐point OD of approximately 40 cm^−1^ which corresponded to 13.8 g_dcw_.L^−1^. Nonetheless, the DO control proved to be unsuccessful in maintaining the dissolved oxygen above the set point without considerable variance. These complications may have arisen from using a single valve to control the gas mix or by the use of two algorithms to specify gas mixture and pressure cycling through peristalsis channels (gas mixture PMW frequency ranged between 0.1 and 3 Hz). The DO control was achieved by Lee et al. 13 over a 3 week continuous fermentation of E. coli. Optical densities of 2 cm^−1^ were achieved corresponding to 0.7 g_dcw_.L^−1^. The dilution rate was typically 0.3 h^−1^ and the turbidostat control variance was less than 1.2%. The gas mixture was varied at 10 Hz.

The real‐time determination of oxygen uptake rate was possible by implementing Equation (2). This is an improvement compared with previous publications where the OUR was calculated by analyzing the data off‐line and not in real time. In Mehta *et al.* 29 the OUR of human hepatocellular carcinoma cells (an adherent cell culture) was determined by modeling oxygen transfer with experimental dissolved oxygen in microchannels of 200 µm height and 300 µm wide. The dissolved oxygen was measured by optics‐based lifetime detection technique.^30^ A different approach was followed by Saito *et al.* 31 where the oxygen concentration was measured by scanning electrochemical microscopy in PDMS microchannels of 200 µm height and 220 µm width. The OUR of *Escherichia coli* (suspension cell culture) was determined by applying logarithmic least‐square methods to the measured dissolved oxygen concentration.

More recently, Peterat *et al.* 15 determined the OUR of *S. cerevisiae* in a vertical microbubble column with a working volume of 70 µL. The OUR was calculated from the variance in DO when the gas flow was on and a quasi‐steady‐state of oxygen concentration occurred, or from a dynamic DO profile when the gas flow is interrupted.

The OUR profiles obtained from three runs overlapped, having an average maximum of 34.3 mmol L^−1^ h^−1^ (Fig. 6(B)). These profiles correspond to an average *k_L_a* of 90 h^−1^. The difference between this value and the 174 h^−1^ measured during oxygen mass transfer characterization can possibly be due to an increase in media viscosity and sedimentation of the *S. cerevisiae*. In fact, there was an observed tendency for sedimentation in particular around the chamber perimeter and tubing (data not shown). The tendency of *S. cerevisiae* to sediment and aggregate may explain the paucity of it being used as a model system in µBRs, despite its high industrial and scientific importance.

An example of full monitoring and control capabilities can be depicted from Fig. 6(C). Clear correlation between the DO profiles and the OD can be observed, as well as the duration of the lag phase (up to 4 h operation) and exponential phase (from 4 h operation onwards). A cell density of 6.7 g_dcw_ L^−1^ was reached in this µBR configuration.

The OD profiles of independent runs showed significant variance. To avoid this, the data could be normalized by inoculum OD or a more robust inoculation procedure devised.

## CONCLUSIONS

An oscillating jet driven monitored and controlled microbioreactor has been developed, in which active mixing was accomplished without on‐chip moving parts. This mixing mechanism was able to achieve mixing times of 7 s and maximum volumetric mass transfer coefficient of *∼*170 h^−1^ in a fluidic assessment assay with beads.

The implemented oxygen and temperature control system proved to be reliable, reproducible and robust allowing controlled growth of *S. cerevisiae* over 30 h, without inducing visible cellular stress or medium evaporation, enabling cell densities of 6.7 g_dcw_ L ^−1^. Oxygen uptake rates were determined from oxygen profiles and *k_L_a*, reaching a maximum of ∼34 mmol L^−1^ h^−1^.

The use of disposable layers and the added capabilities to monitor and control several process parameters make this setup particularly attractive to speed up the development and optimization of fermentation processes. However, improvements are necessary to increase data quality regarding cellular growth and to prevent sedimentation. The incorporation of additional analytical tools (e.g. bio‐luminescence or fluorescence probes) and control of other process parameters, such as pH and glucose, will in the future increase the versatility of the designed platform. Furthermore, to achieve higher cell densities, the platform could be expanded to enable fed‐batch strategies.

## Supporting information

Oxygen Uptake Rate and Dissolved Oxyygen Control
Contamination Clearance Test
Relationship between the solenoid microvalve output and the duty cycle of the oxygen concentration
