# Supplementary material for: Quantification of the oxygen uptake rate in a dissolved oxygen controlled oscillating jet‐driven microbioreactor
Source: J Chem Technol Biotechnol. 2016 Jan 12;91(3):823–31. doi: 10.1002/jctb.4833 (PMC4950047; doi:10.1002/jctb.4833)
Supplement: Supplementary file 1 — Oxygen Uptake Rate and Dissolved Oxyygen Control Contamination Clearance Test Relationship between the solenoid microvalve output and the duty cycle of the oxygen concentration [file JCTB-91-823-s001.docx]

SUPPORTING INFORMATION **-** Kirk et al., Quantification of the oxygen uptake rate in a dissolved oxygen controlled oscillating jet-driven microbioreactor

1. **Oxygen Uptake Rate and Dissolved Oxyygen Control**

The dissolved oygen was controlled by applying a mass balance to the oxygen concentration, according to

where *k_L_a, C*, C_O2_* and *OUR*, are the oxygen volumetric mass transfer coefficient, the liquid oxygen saturation concentration for a given oxygen partial pressure, the dissolved oxygen concentration, and the oxygen uptake rate, respectively. The *C** is a funtion of the oxygen partial pressure given by Henry’s Law

where y, H and x are the oxygen gas phase partial pressure, Henry’s law coefficient and the oxygen liquid phase concentration, respectively. At low concentrations, it can be assumed that *𝑥∝𝐶*, as is *y*, so that the following relationship can be made

The liquid oxygen saturation concentration is the only parameter that can be varied during reactor operation, thus is ideally the one to be controlled. This control is accomplished by changing the ratio between oxygen and nitrogen concentration via a pulse width modulation (PWM) valve (LHDA1211111H, Lee Co., USA). The use of PWM valves to control gas mixes is a common practice in the field of microbioreactors {Lee:2006bn}. The PWM valve is controlled by the output of a bespoke LabVIEW (National Instruments, USA) PID algorithm

$$Output=P\cdot E+I\cdot\int_{t0}^{tf} Edt+D\frac{dE}{dt}$$

where *P, I, D* and *E* are the proportional, the integral and derivative values and the difference between the oxygen concentration and the estabelished set point, respectively. The values of P (0.06), I (0.012), and D (0) where optimised with various tuning algorithms, including the LabVIEW auto-tuner.

During operation, if *C** exceeds *C_O2_* the output for the PWM valve is to open the “normally closed” port (connected to air supply) to increase oxygen concentration in the headspace above the microbioreactor aeration membrane and close the “normally open” port (connected to nitrogen supply). Conversely, if*C_O2_* exceeds C*, then the output to the PWM valve is to close the “normally closed” port and to open the “normally open” port. This allows the PID algorithm to increase or decrease *C_O2_* until the set point has been reached (or until small oscillations of acceptable amplitude about the set point have been reached).

Since the dissolved oxygen (DO) can be monitored in real time, then OUR can be calculated in real time from

At steady state under DO control the oxygen concentration differential can be removed. Since the DO control is *via* a PWM gas mix scheme, *C^*^* can be substituted by the product of *C^*^* and the PWM duty cycle (DC). i.e.

The duty cycle expresses how long the PWM valve is open to the air supply versus the full operation time.

1. **Contamination Clearance Test**

Two PDMS chips were incubated for 24 hours at 37ºC in YPD10 medium. One PDMS chip was sterilized with 70% (v/v) ethanol before being placed in a 50 mL Falcon tubes containing YPD10 medium. A non-sterilized PDMS chip was put in YPD10 medium (positive control) in a separate Falcon tube. A third Falcon Tubes contained only YPD10 medium (negative control). The figure below shows the Falcon tubes with the their respective final OD600 measurements. From left to right: negative control, sterilised PDMS chip, positive control.


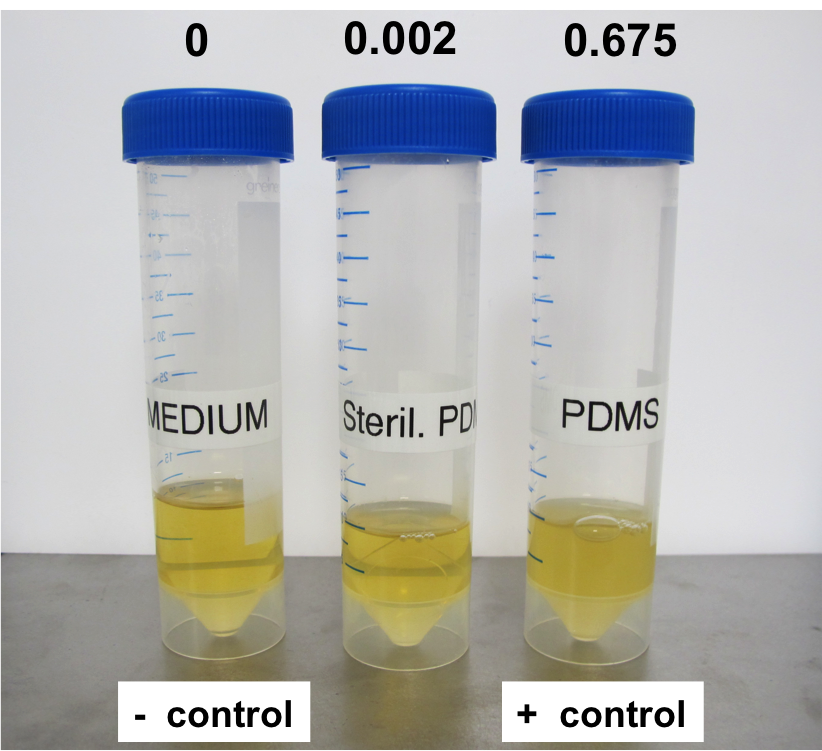


1. **Relationship between the solenoid microvalve output and the duty cycle of the oxygen concentration**


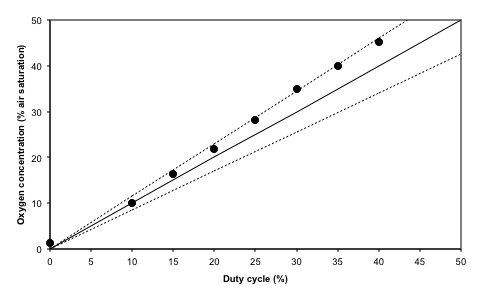


Relationship between the solenoid microvalve output and the duty cycle of the oxygen concentration between zero and 40% air saturation. Air was fed to the normally closed port and nitrogen to the normally open port. Dashed lines correspond to a deviation of 15% from the center points (closed line).
